# Proteomics identifies differences in fibrotic potential of extracellular vesicles from human tendon and muscle fibroblasts

**DOI:** 10.1186/s12964-020-00669-9

**Published:** 2020-11-04

**Authors:** Ching-Yan Chloé Yeung, Erwin M. Schoof, Michal Tamáš, Abigail L. Mackey, Michael Kjaer

**Affiliations:** 1 Institute of Sports Medicine Copenhagen, Bispebjerg Hospital, Nielsine Nielsens Vej 11, Building 8, Copenhagen, NV 2400 Denmark; 2 Center for Healthy Aging, University of Copenhagen, Copenhagen, Denmark; 3 Proteomics Core, Technical University of Denmark, Kongens Lyngby, Denmark; 4 Department of Biomedical Sciences, Faculty of Health and Medical Sciences, University of Copenhagen, Copenhagen, Denmark

**Keywords:** Extracellular vesicle, Musculoskeletal tissue, Mass spectrometry, Extracellular matrix

## Abstract

**Background:**

Fibroblasts are the powerhouses responsible for the production and assembly of extracellular matrix (ECM). Their activity needs to be tightly controlled especially within the musculoskeletal system, where changes to ECM composition affect force transmission and mechanical loading that are required for effective movement of the body. Extracellular vesicles (EVs) are a mode of cell-cell communication within and between tissues, which has been largely characterised in cancer. However, it is unclear what the role of healthy fibroblast-derived EVs is during tissue homeostasis.

**Methods:**

Here, we performed proteomic analysis of small EVs derived from primary human muscle and tendon cells to identify the potential functions of healthy fibroblast-derived EVs.

**Results:**

Mass spectrometry-based proteomics revealed comprehensive profiles for small EVs released from healthy human fibroblasts from different tissues. We found that fibroblast-derived EVs were more similar than EVs from differentiating myoblasts, but there were significant differences between tendon fibroblast and muscle fibroblast EVs. Small EVs from tendon fibroblasts contained higher levels of proteins that support ECM synthesis, including TGFβ1, and muscle fibroblast EVs contained proteins that support myofiber function and components of the skeletal muscle matrix.

**Conclusions:**

Our data demonstrates a marked heterogeneity among healthy fibroblast-derived EVs, indicating shared tasks between EVs of skeletal muscle myoblasts and fibroblasts, whereas tendon fibroblast EVs could play a fibrotic role in human tendon tissue. These findings suggest an important role for EVs in tissue homeostasis of both tendon and skeletal muscle in humans.

Video abstract

**Supplementary information:**

**Supplementary information** accompanies this paper at 10.1186/s12964-020-00669-9.

## Background

Fibroblasts are cells responsible for producing extracellular matrix (ECM) and their activity is tightly regulated. For examples, too much or too little ECM causes fibrosis or tissue frailty, respectively, and changes to the ECM composition will affect both the mechanical properties and the biochemistry of the tissue. A relatively new mechanism by which fibroblasts can be regulated is via extracellular vesicles (EVs), which are cell-released lipid membrane encapsulated particles. The two most studied subtypes of EVs are exosomes, which are 50–150 nm vesicles that are derived from the endosomal pathway and microvesicles (sometimes referred to as ectosomes), which are from 100 nm up to 1 μm in size and are formed by direct budding of the plasma membrane [1, 2]. Although they have different routes to their formation there are no specific protein markers to differentiate them [2, 3]; Many marker proteins for exosomes are also present in microvesicles [2], which include tetraspannins (e.g. CD81), heat shock proteins, components of the endosomal sorting complexes required for transport (ESCRT), integrins and regulators of intracellular trafficking (e.g. Ras-associated binding proteins, annexins and clathrins). The molecular content of exosomes is, however, highly specific to the cell of origin and can be passed on to other cells as part of intra- and inter-tissue communication [4–7]. How exosomes and other EVs from healthy fibroblasts regulate tissue homeostasis is unknown and the lack of biomarkers makes them difficult to study.

Multiple studies have demonstrated that exosomes and microvesicles are able to dock and fuse with cells to deliver functional protein cargo, as well as micro RNAs and messenger RNAs [2, 3, 5]. The most studied examples of exosome-mediated crosstalk with fibroblasts are in tumour growth and metastasis [8, 9]. Exosomes produced by cancer cells activate stromal fibroblasts to become cancer-associated fibroblasts (CAFs) and exosomes released from CAFs induce metastatic properties in cancer cells lines and have also been demonstrated to reprogram stromal fibroblasts in the pre-metastatic niche [6, 10, 11]. Induction of the pro-tumour progression phenotype in normal stromal fibroblasts is attributed to transforming growth factor β1 (TGFβ1) that is localised inside CAF exosomes [6, 11]. We hypothesised that EVs from healthy fibroblasts also regulates the ECM during tissue homeostasis and they do so via their tissue-specific, functional cargo.

In the musculoskeletal system tendon fibroblasts make large amounts of type I collagen whereas muscle fibroblasts make more type III than type I collagen, and in smaller amounts than tendon [12, 13]. Treatment of tenocytes in a number of in vitro and in vivo experimental models with EVs from various sources, e.g. plasma [14], adipose stem cells [15], tendon progenitors [16], macrophages [17], have demonstrated that signalling through EVs can modulate the expression of genes that regulate ECM synthesis and remodelling. Exosomes derived from tendon cells have also been reported [18, 19]; and one study showed that tendon cell-derived exosomes induce the expression of tenogenic genes in bone marrow-derived mesenchymal stem cells in a TGFβ-dependent manner [19]. Compared to tendon, muscle is a more complex tissue with more than one cell type. Collagen synthesis by muscle fibroblasts is tightly regulated and exosomes derived from satellite cells play a role in preventing fibrosis in healthy muscle tissues [20]. Interestingly, tendon rupture in humans and animal models causes an increase of type I collagen in the adjacent muscle tissue, which correlates to increased muscle stiffness and decreased muscle function [12, 21–26]. Establishing the biochemistry of healthy fibroblast EVs may be a crucial first step in understanding their role when tissues are damaged.

Exosomes and other EVs have great potential as a non-invasive source of biomarkers for disease detection and monitoring as they can be isolated from various bodily fluids in addition to blood, including urine, saliva, breast milk and sweat [7, 27, 28]. In order to determine the health status of a specific tissue from a pool of EVs derived from all the tissues in the body, we must be able to isolate tissue-specific exosomes and the identification of membrane-localised proteins can aid the capture of EVs for targeted analyses. Recent studies have demonstrated that EV surface proteins bear characteristics of their tissue of origin and these EVs can be captured from bodily fluids using antibody-based assays targeting these proteins [29, 30]. Proteomic profiling of EVs is a novel and sensitive approach to increase understanding of EV function and its use has been successful in unravelling their role in cancer [31]. A proteomic approach will also reveal potential protein biomarkers for isolating tissue-specific EVs. In this study, we took a proteomics approach to investigate and compare the proteome of small EVs isolated from the major cell populations in human tendon and muscle to elucidate key molecules and understand how homeostasis of the different ECMs in these two tissues is regulated.

## Methods

### Ethics

Informed consent was obtained from all tissue donors (ethics approval H-3-2010-070 by the Regional Ethical Committee for the Hospital Region of Greater Copenhagen, in accordance with the Declaration of Helsinki II). The study was reported to the Danish register (Datatilsynet) and was performed in accordance with Danish law (Lov om behandling af personoplysninger).

### Human tissues

Human tissue was obtained in connection with anterior cruciate ligament reconstruction surgery. Gracilis and semitendinosus tendon and muscle tissues were harvested under general anaesthetic. After the surgeon had obtained the tendon required for the reconstruction, any waste tissues were transferred to ice-cold PBS containing 50 U/ml penicillin and 50 μg/ml streptomycin (Thermo Fisher Scientific). Tissues from five biological samples (2 females, 3 males) with a mean age 29.6 ± 7.2 (standard deviation (SD)) years old were used (Supplementary Figure 1a and 1b). Muscle fibroblasts derived from one of the samples did not proliferate enough and so they were not included in the study.

### Tendon fibroblasts

Tendon tissues that were cleaned of muscle tissue were cut into small pieces and incubated overnight in 400 U/ml collagenase type 2 (Worthington Biochemical Corporation) prepared in DMEM/F-12 (Thermo Fisher Scientific) supplemented with 20% FCS (BioWest) and 50 U/ml penicillin and 50 μg/ml streptomycin at 37 °C in 5% CO_2_. After, cells were strained through a 70 μm filter (BD Biosciences) and centrifuged for 6 min at 600 x g, washed with PBS, pelleted again and then resuspended in medium. Tendon fibroblasts were cultured in DMEM/F-12 medium supplemented with 10% FCS and 50 U/ml penicillin and 50 μg/ml streptomycin at 37 °C in 5% CO_2_.

### Myoblasts and muscle fibroblasts

Muscle tissues were cut into small pieces and incubated for 1 h in 2 mg/ml collagenase B (Roche), 2 mg/ml dispase II (Roche) in Skeletal Muscle Basal Medium (Promocell) at 37 °C in 5% CO_2_, with agitation by pipetting every 15 min. After, cells were strained through a 70 μm filter and centrifuged for 6 min at 600 x g, washed with PBS, pelleted again and then resuspended in medium. Muscle tissue cells were cultured for 1 week in Human Skeletal Growth Medium (Promocell) supplemented with 15% FCS, 2 mM L-glutamine, 20 U/ml penicillin and 100 μg/ml streptomycin at 37 °C in 5% CO_2_. After, CD56+ myogenic cells (myoblasts) were sorted from CD56- non-myogenic cells (muscle fibroblasts) as described previously [32]. In brief, muscle cells were treated for a maximum of 2 min at 37 °C in 5% CO_2_ with trypsin EDTA solution C (Biological Industries) diluted 1:2 in PBS. Detached cells were pelleted, washed in PBS and pelleted again. The cell pellet was resuspended in 170 μl MACS Separation Buffer (Miltenyi Biotec) and 35 μl CD56 magnetic beads (Human CD56 MicroBead Kit; Miltenyi Biotec) and incubated for 15 min at 4 °C. After, cells were centrifuged for 6 min at 600 x g, resuspended in MACS Separation Buffer and passed through a 30 μm pre-separation filter into a large cell column attached to a MiniMACS Separator (all from Miltenyi Biotec) following the manufacturer’s protocol. Both CD56- muscle fibroblasts and CD56+ myoblasts were collected and cultured separately in DMEM/F-12 medium supplemented with 10% FCS and 50 U/ml penicillin and 50 μg/ml streptomycin and in Human Skeletal Growth Medium supplemented with 15% FCS, 2 mM L-glutamine, 20 U/ml penicillin and 100 μg/ml streptomycin, respectively, at 37 °C in 5% CO_2_. Before EV isolation, myoblasts were differentiated by culturing for 4 days in Skeletal Muscle Basal Medium supplemented with 2 mM L-glutamine, 20 U/ml penicillin and 100 μg/ml streptomycin, at 37 °C in 5% CO_2_.

### Small extracellular vesicle isolation

For each sample, cells ~ 80% confluency from one T175 flask were used for small EV isolation. Cells were cultured without serum, in DMEM/F-12 supplemented with 0.035% sodium bicarbonate, 10 mM HEPES, 1x GlutaMAX supplement and 50 U/ml penicillin and 50 μg/ml streptomycin at 37 °C in 5% CO_2_. After 16 h, the conditioned medium was collected. The conditioned media was ultra-filtered through 0.22 μm filter (Satorius) to remove any intact cells, contaminating microvesicles and apoptotic bodies, and then centrifuged at 2000 x g for 20 min at 4 °C to remove any remaining cell debris. The supernatant was further centrifuged at 4566 x g for 1 h at 4 °C to remove any remaining microvesicles. The supernatant was ultracentrifuged in 5-ml polypropylene centrifuge tubes (Beckman Coulter) at 100000 x g for 2 h at 4 °C using an SW55Ti rotor and Optima L-80 XP Ultracentrifuge (Beckman Coulter). The pellet containing crude EV extract was resuspended in 40 μl PBS containing protease inhibitor cocktail (Roche). The EV extract was mixed with 4 ml BioUltra PBS (Sigma-Aldrich) and ultracentrifuged in 5-ml polypropylene centrifuge tubes at 100000 x g for 2 h at 4 °C. The pellet of small EVs was then resuspended in 20 μl 6 M guanidine hydrochloride, 10 mM Tris (2-carboxyethyl) phosphine hydrochloride, 40 mM 2-chloroacetamide, 100 mM Tris pH 8.5 for mass spectrometry analysis. Samples were stored at -80 °C.

### Transmission electron microscopy

For TEM analysis of EVs, EV pellets were re-suspended and fixed in 2% glutaraldehyde in 100 mM phosphate buffer. Fixed EVs were mounted on to glow-discharged copper grids (Agar Scientific) coated with a continuous carbon film, then stained with 1% (w/v) uranyl acetate (Sigma Aldrich) in ddH_2_O for 1 min at RT, and washed with ddH_2_O. Grids were examined with a CM 100 TWIN (Philips) fitted with a 2 k × 2 k side-mounted TEM CCD camera (Olympus Veleta). EV diameter measurements were made using FIJI. Median diameter was calculated from at least 300 measurements per sample, the average and SD of median EV diameters were calculated from 3 biological samples.

### Tryptic digestion of small EV proteins

Protein concentrations were determined by Quick Start Bradford Protein Assay (Bio-Rad). In 1.5 ml low-bind eppendorf tubes proteins (< 5 μg in 20 μl) were digested with 10% acetonitrile in 50 mM HEPES pH 8.5 (40 μl) containing Lys-C protease (20 ng/μg protein) at 37 °C, 600 rpm for 3 h and with additional trypsin (10 ng/μg protein) and 10% acetonitrile in 50 mM HEPES pH 8.5 (140 μl) for 16 h at 37 °C, 600 rpm. After, samples were acidified with equal volume of 2% trifluoroacetic acid (200 μl) and vortexed. Acidified samples (all 400 μl) were then run through equilibrated stage tips containing two Empore C18 filters (3 M) prepared in 200-μl pipette tips. Stage tips were centrifuged at 3000 rpm until the sample had filtered through. Peptides were eluted in 30 μl 40% acetonitrile and 0.1% formic acid into 1.5 ml low-bind eppendorf tubes and dehydrated with a speed vac at 65 °C for ~ 60 min. Dehydrated samples were then resuspended in 12 μl 2% acetonitrile and 1% trifluoroacetic acid containing indexed retention time peptides (iRT peptides at 1:500, Biognosys AG) and transferred to 0.5 ml low-bind eppendorf tubes. Peptide concentrations were measured using a DS-11 FX+ spectrophotometer (DeNovix).

### Liquid chromatography-mass spectrometry (LC-MS)

For each sample, 50 ng peptides were loaded onto a 2-cm C18 trap column (Thermo Fisher Scientific), connected in-line to a 50-cm C18 reverse-phase analytical column (Easy-Spray ES803 LC column, Thermo Fisher Scientific) using 0.1% formic acid in water at 4 μl/min, using the Easy-nLC 1200 high-performance liquid chromatography system (Thermo Fisher Scientific), and the column oven operating at 45 °C. Peptides were eluted over a 140-min gradient ranging from 6 to 60% of 80% acetonitrile, 0.1% formic acid at 250 nl/min, and the Orbitrap Fusion instrument (Thermo Fisher Scientific) was run in a data-dependent-MS/MS (DD-MS2) top speed method. Full MS spectra were collected at a resolution of 120,000, with an AGC target of 4 × 10^5^ or maximum injection time of 50 ms and a scan range of 400–1500 m/z. The MS2 spectra were obtained in the ion trap operating at rapid speed, with an AGC target value of 1 × 10^4^ or maximum injection time of 35 ms, a normalised higher-energy collisional dissociation (HCD) collision energy of 30 and an intensity threshold of 1.7e^4^. Dynamic exclusion was set to 60 s, and ions with a charge state < 2, > 7 or unknown were excluded. MS performance was verified for consistency by running complex cell lysate quality control standards, and chromatography was monitored to check for reproducibility.

### Label-free quantitative proteomics analysis

The mass spectrometry data have been deposited to the ProteomeXchange Consortium via the PRIDE partner repository (http://www.ebi.ac.uk/pride/archive/) with the data set identifier PXD018233. The mass spectrometry raw files were analysed using Proteome Discoverer 2.4 and can be found in Supplementary Data 1. Label-free quantitation (LFQ) was enabled in the processing and consensus steps, and spectra were matched against the *Homo sapiens* database obtained from Uniprot. Dynamic modifications were set as oxidation (M), deamidation (N,Q) and acetyl on protein N-termini. Cysteine carbamidomethyl was set as a static modification. All results were filtered to a 1% false discovery rate (FDR), and protein quantitation done using the built-in Minora Feature Detector. Proteins suggest by the Minimal Information of Studies for EVs 2018 [33] were used for protein content-based EV characterisation.

### Statistical analysis of LC-MS data

The normalised protein intensities generated by LC-MS were analysed using the R-based integrated web application Differential Expression and Pathway version 0.90 (iDEP) [34]. In the interest of identifying fibroblast EV-enriched proteins, only those detected in at least 2 biological replicates of small EVs derived from tendon fibroblasts (TenX) and small EVs derived from muscle fibroblasts (FibX) samples were further analysed (612 proteins). We did not find that removing proteins affected the distribution pattern of TenX and FibX samples. Supplementary Data 2 contains the customised R code for the iDEP workflow. Supplementary Data 3 contains the log transformed protein intensities with missing values filled in by imputation of the median intensity for the protein within the sample group. iDEP-generated values for heatmaps can be found in Supplementary Data 4 and 5. Supplementary Data 6 contains the results from the DESeq2 (an iDEP package), using a threshold of false discovery rate (FDR) *p* < 0.1 and fold-change > ± 2. Functional enrichment analysis was performed on high abundance proteins, e.g. by combining proteins identified as high in one type of small EV, when compared to the other two types of small EVs using the online tool DAVID version 6.7 [35] and the resultant enrichment clusters are contained in Supplementary Data 7 and 8. Venny version 2.1 (BioinfoGP, Spanish National Biotechnology Centre) was used to identify common and unique proteins between groups and the output can be found in Supplementary Data 9. Statistical significance indicated in figures (*p < 0.05, **p < 0.01, ***p < 0.001 and ****p < 0.0001) was derived from the DESeq2 analysis (see Supplementary Data 6 for all adjusted *p* values).

## Results

### Comparison of proteins from human EVs

We derived and expanded sufficient number of tendon fibroblasts, myoblasts (CD56+) and muscle fibroblasts (CD56−) cells from four biological samples but only tendon fibroblasts and myoblasts from sample 5 (Supplementary Figure 1a and 1b). Small EVs isolated from conditioned media from tendon fibroblasts (TenX), differentiating myoblasts (MyoX) and muscle fibroblasts (FibX) were analysed by transmission electron microscopy (TEM). EV preparations from these three cell types showed the presence of membrane-bound vesicles with median diameters of approximately 30–50 nm, where MyoX were the largest and FibX were the smallest (Supplementary Figure 1c and 1d). EV preparations were digested with trypsin and the peptides were prepared for LC-MS. The raw LC-MS data were searched against a human database and were mapped to 1850 proteins and of these, 1179 were supported by at least two unique peptides and of these, 612 proteins were detected in at least two of the five TenX samples and two of the four FibX samples (Supplementary Figure 2a). Distribution of the transformed data showed that all five TenX samples contained a narrower distribution of protein abundances and contained fewer of the low abundance proteins compared to MyoX or FibX samples (Supplementary Figure 2b and 2c). FibX samples also showed more variation in protein abundance in the four replicates than the MyoX or TenX samples.

**Fig. 1:**
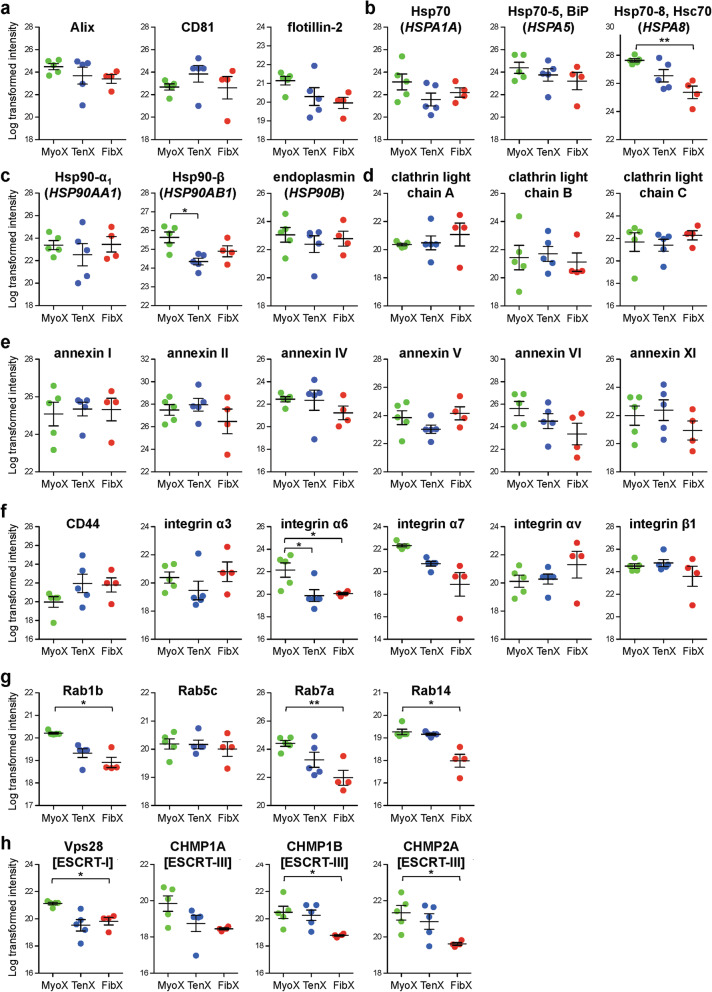
Presence of exosome-enriched proteins in small EVs derived from tendon and muscle tissue cells. **a-g** Log transformed intensity of **a** exosome-enriched, **b** HSP70, **c** HSP90, **d** clathrin, **e** annexin, **f** cell surface receptor, **g** Ras-associated binding (Rab), and **h** endosomal sorting complexes required for transport (ESCRT) proteins detected in EVs isolated from differentiating myoblasts (MyoX), tendon fibroblasts (TenX) and muscle fibroblasts (FibX). *p < 0.05, **p < 0.01. See Supplementary Data 3 for transformed intensity values used for statistical analysis. See Supplementary Data 6 for all adjusted *p*-values from DESeq2 analysis

The proteome of the EV preparations contained proteins known to be enriched in EVs and proteins often co-isolated with EVs (Supplementary Figure 3). Commonly used exosome marker proteins, Alix (ALG-2-interacting protein X), tetraspanin CD81, and flotillin-2 were detected in all EV samples (Fig. 1a). Other EV-enriched proteins, chaperones heat shock protein (Hsp) 70 (Fig. 1b), Hsp90 (Fig. 1c), clathrins (Fig. 1d), annexins (Fig. 1e), cell-surface receptors (Fig. 1f), Ras-associated binding (Rab) proteins (Fig. 1g) and endosomal sorting complexes required for transport (ESCRT) proteins (Fig. 1h) were also present the LC-MS data set. There were no significant differences in the abundance of these exosomal markers except for heatshock protein 70 (Hsp70) (Hsc70; *HSPA8*) (Fig. 1b), Hsp90-β (Fig. 1c), integrin α6 (Fig. 1f), Rab1b, Rab7a and Rab14 (Fig. 1g), Vps28, CHMP1B (charged multivesicular body protein 1B) and CHMP2A (Fig. 1h), the abundance of which were significantly higher in MyoX.

**Fig. 2:**
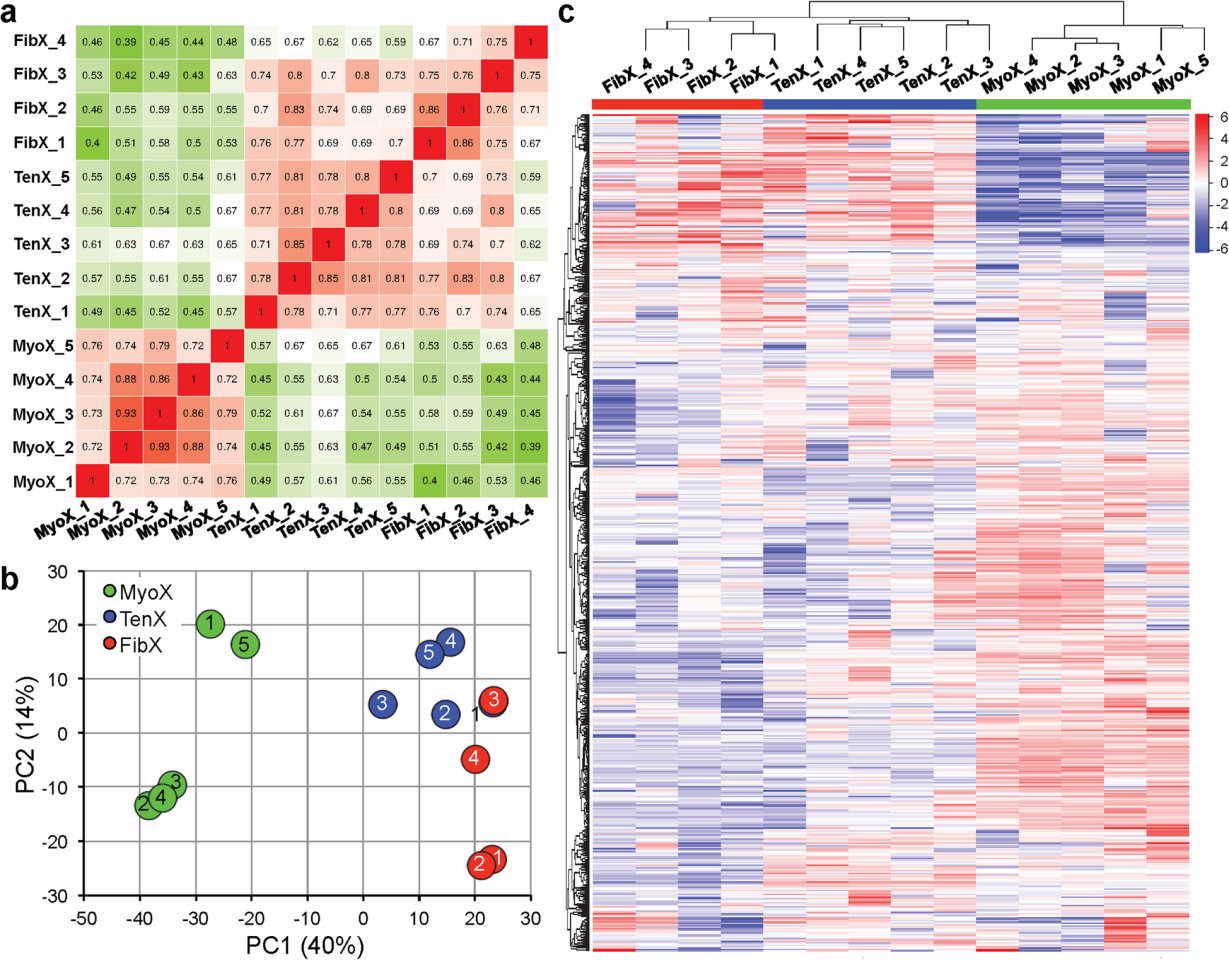
Variability in small EV samples. **a** Heatmap of Pearson correlation coefficient (r) for pairwise comparison of each MyoX, TenX and FibX sample. Each square represents a single comparison with the intensity of colour denoting the r-value according to the number within. High r-values (red squares) denote high correlation and green squares denote low correlation between samples, respectively. **b** Principal component analysis (PCA) showing the first two principle components (PC1 and PC2). Numbers inside the circles indicate the sample numbers. **c** Heatmap showing hierarchical clustering of samples based on their protein abundances ranked by SD. See Supplementary Figure 4 for heatmap values

Pearson’s correlation coefficients of individual TenX, MyoX and FibX biological replicates showed strong correlation within MyoX samples and between TenX and FibX samples (Fig. 2a). This grouping of fibroblast-derived small EVs was also observed when principal component analysis (PCA) was performed. The first PC identified that the largest variance in the data set, which accounted for 40% of the variability, was between MyoX and TenX/FibX samples, and PC2 identified variances between TenX and FibX samples but there was overlap of TenX sample 1 and FibX sample 3 (Fig. 2b). iDEP program ranked the proteins by their standard deviation across all the samples by hierarchical clustering. The resultant heatmap further highlighted the similarities in the protein abundance pattern between TenX and FibX samples (Fig. 2c, Supplementary Data 4).

### Functional annotation of proteins enriched in MyoX, TenX and FibX

**Fig. 3:**
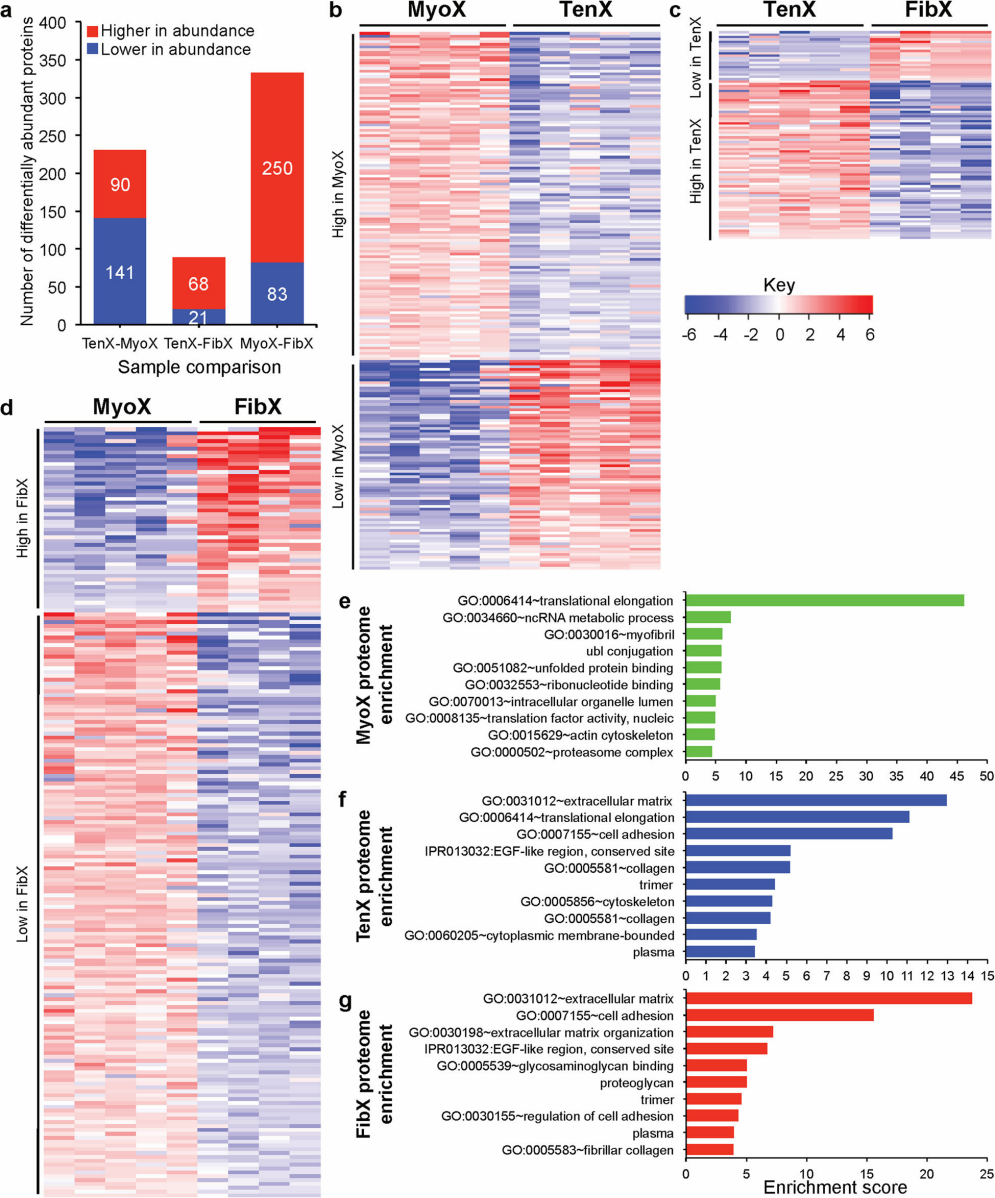
Enriched proteins in MyoX, TenX and FibX. **a** Number of proteins with over ±2-fold mean differential abundance in small EV samples, when cells of origin were compared (FDR *p* < 0.1). **b-d** Heatmaps showing the abundance pattern across individual samples of proteins with over ± ≥ 2-fold mean differential abundance in **b** TenX compared to MyoX, **c** TenX compared to FibX, and **d** MyoX compared to FibX. The range of fold change is represented by the colour key. See Supplementary Data 6 for heatmap values. **e-g** Top 10 significantly enriched terms, ranked by their enrichment scores, that were overrepresented in highly abundant proteins of **e** MyoX (391 proteins), **f** TenX (185 proteins), and **g** FibX (104 proteins). See Supplementary Data 7 for the full enrichment analysis results

Differentially abundant proteins with a greater than ±2-fold mean and FDR *p* < 0.1 were identified. As expected, a comparison of TenX and FibX samples yielded the fewest differentially abundant proteins (89 proteins; Fig. 3a and c, Supplementary Data 6). Surprisingly, the MyoX and FibX comparison identified more differentially abundant proteins than TenX and MyoX comparison (333 and 231 proteins, respectively; Fig. 3a, b and d, Supplementary Data 6). Functional enrichment analysis showed that MyoX contained a high abundance of proteins that were overrepresented in translation, RNA processing, which included many components of 60S and 40S ribosomal subunits, chaperones and over 80 RNA-binding proteins (Fig. 3e, see Supplementary Data 7 for protein identifications) and unsurprisingly there was an overrepresentation of myofibril proteins in MyoX. In TenX there was an enrichment of proteins that were of the ECM including collagens and proteoglycans, and similar to MyoX, contained many of the same translation components (Fig. 3f, see Supplementary Data 7 for protein identifications). Surprisingly, FibX were not enriched in proteins that support translation but similar to TenX, they were enriched in ECM proteins (Fig. 3g, see Supplementary Data 7 for protein identifications).

### Comparison of ECM proteins of fibroblast EVs

**Fig. 4:**
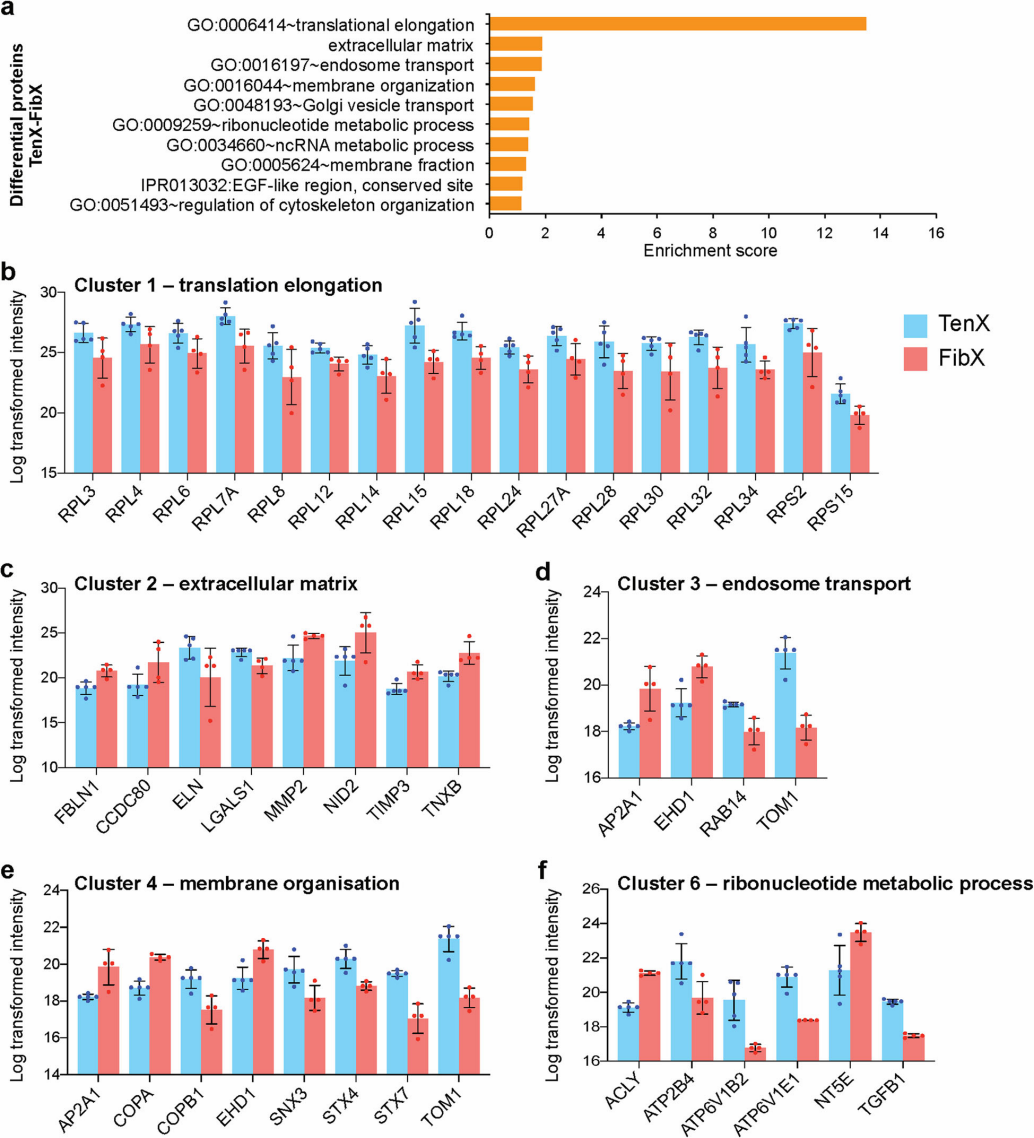
Functional enrichment analysis of proteins with differential abundance between TenX and FibX. **a** Top 10 significantly enriched terms, ranked by their enrichment scores that were overrepresented in the proteins differentially abundant in a comparison of TenX and FibX proteins (89 proteins). **b-e** Log transformed intensity of proteins in **b** cluster 1, **c** cluster 2, **d** clusters 3, **e** cluster 4 and **f** cluster 6. See Supplementary Data 8 for the full enrichment analysis results

To elucidate the different functions of TenX and FibX we performed functional enrichment analysis on the 89 significantly differentially abundant proteins. The top enrichment term was translation elongation, followed by ECM, and terms that contained vesicle (endosome, Golgi) transport/membrane proteins and RNA metabolism proteins, and cytoskeletal organisation (Fig. 4a, see Supplementary Data 8). All translation proteins present under the enrichment term were more abundant in TenX than in FibX and included two 40S ribosomal subunits and 15 60S ribosomal (Fig. 4b). There were differences in the abundance of specific ECM proteins; fibulin-1 (FBLN1), matrix metalloproteinase 2 (MMP2), nidogen-2, metalloproteinase inhibitor 3 (TIMP3) and tenascin-X (TNXB) were present at higher abundance in FibX than in TenX (Fig. 4c). Membrane organisation proteins involved in transport in endosomes and Golgi vesicles were also identified as differentially enriched between TenX and FibX (Fig. 4d and e). Cation-transporting ATPases (ATP2B4, ATP6V1B2 and ATP6V1E1) under the enrichment term ribonucleotide metabolism process were also more abundant in TenX than in FibX (Fig. 4f).

### Identification of fibroblast-specific small EV proteins

**Fig. 5:**
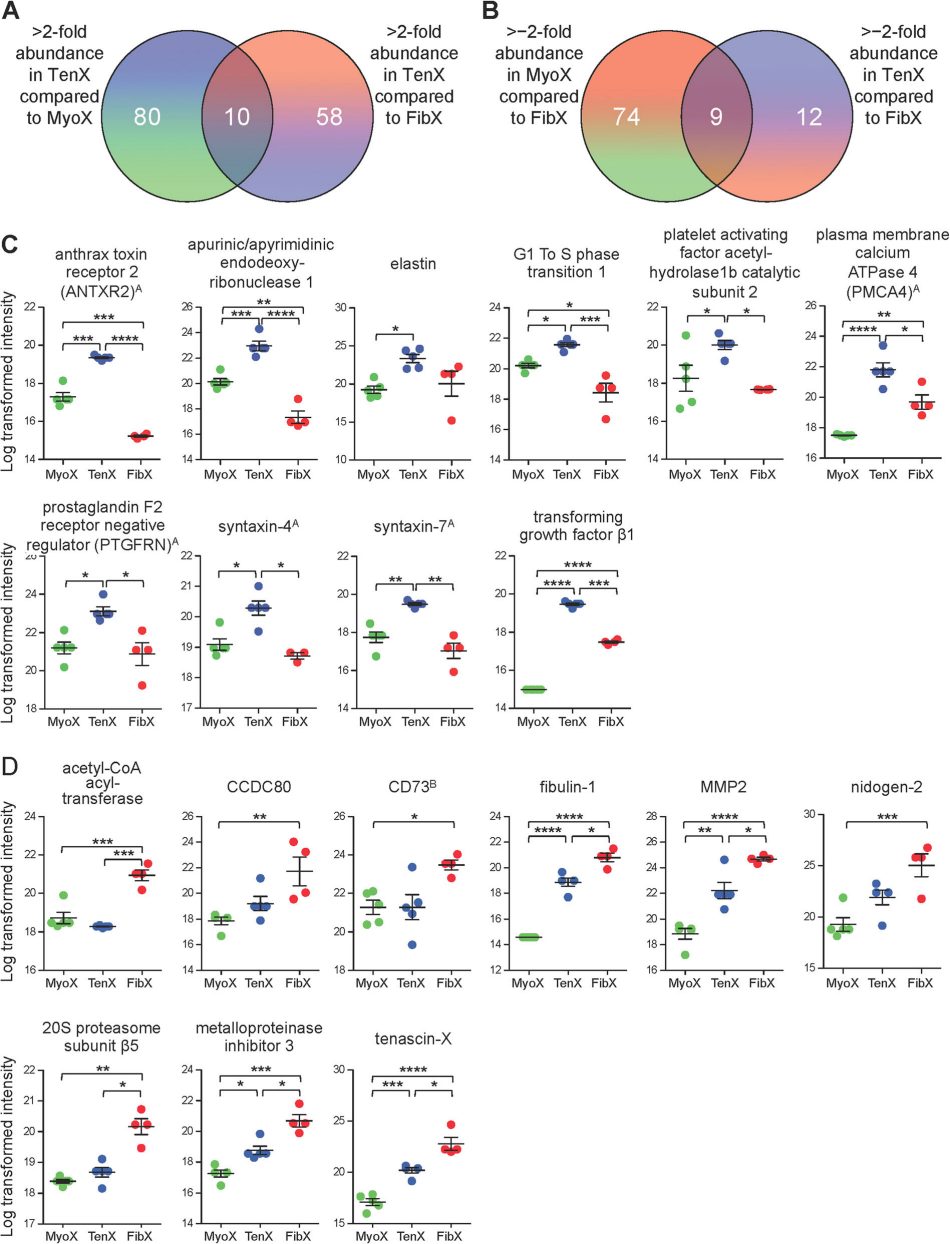
Identification of tendon EV-enriched proteins. **a** Venn diagram showing the number of proteins with > 2-fold mean differential abundance in TenX when compared to MyoX (90 proteins) and in TenX when compared to FibX (68 proteins), with 10 proteins in common. **b** Venn diagram showing the number of proteins with > − 2-fold mean differential abundance in TenX when compared to FibX (21 proteins) and in MyoX when compared to FibX (83 proteins), with 9 proteins in common. See Supplementary Data 9 for full list of proteins (FDR < 0.01). **c** Plots showing the log transformed intensities of the 10 commonly over abundant TenX proteins. ^A^Membrane-localised proteins: ANTXR2, PMCA4 (*ATP2B4*), PTGFRN, syntaxin-4 and syntaxin-7. **d** Plots showing the log transformed intensities of the 9 commonly over abundant FibX proteins. ^B^Membrane-localised protein: CD73 (*NT5E*). *p < 0.05, **p < 0.01, ***p < 0.001 and ****p < 0.0001. See Supplementary Data 6 for all adjusted p-values from DESeq2 analysis

To identify unique TenX-enriched proteins, those that were significantly higher in abundance from TenX-MyoX and TenX-FibX comparisons were compared and 10 proteins were found to be uniquely abundant in TenX (FDR < 0.1; Fig. 5a, see Supplementary Data 9). Of these, anthrax toxin receptor 2 (ANTXR2), apurinic/apyrimidinic endodeoxyribonuclease 1 (APEX1), G1 to S phase transition 1 (GSPT1), platelet activating factor acetylhydrolase 1b catalytic subunit 2 (PAFAH1B2), plasma membrane Ca^2^^+^−ATPase 4 (PMCA4; *ATP2B4*), prostaglandin F2 receptor negative regulator (PTGFRN), syntaxin-4, syntaxin-7 and TGFβ1 proteins were significantly higher in abundance in TenX than in MyoX or FibX (Fig. 5c). We performed the same analysis for the identification of FibX-enriched proteins and found 9 proteins were uniquely abundant in FibX (Fig. 5b, see Supplementary Data 9). Of these, acetyl-CoA acetyl-transferase (HADHB), fibulin-1, MMP2, proteasome 20S subunit β5 (PSMB5), metalloproteinase inhibitor 3 (TIMP3) and tenascin-X were significantly higher in abundance in FibX than in MyoX or TenX (Fig. 5d).

## Discussion

**Fig. 6:**
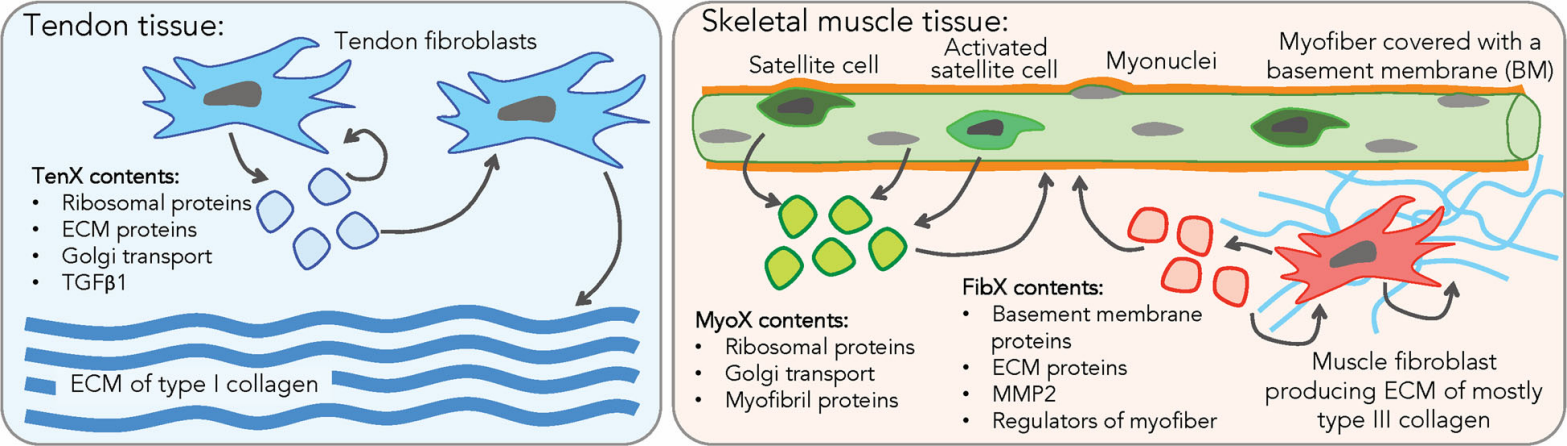
Summary of proposed functional roles of small EVs from human tendon and muscle cells in tissue homeostasis. In tendon, tendon fibroblasts produce EVs (TenX), which act through autocrine or paracrine signalling to induce type I collagen expression, synthesis and assembly into the ECM. In skeletal muscle, EVs produced by myoblast progenitors (satellite cells, activated satellite cells) and myofibers (MyoX) regulate myoblast differentiation and myofibrillogenesis. There are EVs that are produced by muscle fibroblasts (FibX), which also help regulate myoblast differentiation but they also regulate the homeostasis of the basement membrane enveloping the myofibers and the fine collagen network of the endomysium

In this study, we identified key functional differences of small EVs produced by healthy fibroblasts isolated from human musculoskeletal tissues. By performing label-free quantitative LC-MS-based proteomics analysis we have, in an unprecedented manner, established three different proteome profiles of small EVs isolated from primary cultures of tendon fibroblasts, muscle fibroblasts and differentiating myoblasts. We found a high abundance of proteins that support substantial ECM synthesis in tendon fibroblasts EVs but not in muscle fibroblast EVs. In differentiating myoblast EVs a high myofibrillar synthesis was indicated, and in skeletal muscle fibroblast EV proteins supporting myoblast differentiation and the skeletal muscle ECM were present (Fig. 6).

Proteome studies on EVs have focused on cancer, in which high numbers of EVs containing many more proteins are produced [31, 36, 37]. We identified over 1000 proteins in small EVs purified from conditioned media and confirmed the presence of EV marker proteins. No one has to our knowledge previously performed EV proteomics on human tendon tissue. The only other tendon fibroblast EV proteome data published is of porcine tendon exosomes, where only 199 proteins were identified [18], making the current TenX proteome profile the most comprehensive to date. MyoX contained significantly more of some of EV marker proteins, which was expected as Hsc70 and Hsp90-β upregulation are required during myoblast differentiation [38–40], and the presence of these chaperones might be a mechanism of autocrine or paracrine signalling. Integrin α6 is a laminin receptor and their enrichment in MyoX is reflective of the basement membrane adhesion profile of differentiating myoblasts [41], and ESCRT-III molecules are enriched in myoblasts as they are responsible for the shedding of injured membranes [42]. MyoX were also enriched in proteins required for translation elongation, and this enrichment is consistent with previously published biochemical contents of differentiating human myoblasts-derived exosomes and microvesicles [43]. Taken together, our data suggest that the main role of MyoX is to regulate myoblast differentiation and/or myofiber homeostasis (Fig. 6).

We used iDEP, an R-based web application designed to easily analyse transcriptomic and proteomic data [34]. The analysis revealed that the largest source of variability was between fibroblast- and non-fibroblast-derived EVs and the second largest source of variance was between TenX and FibX proteome profiles. So, despite the fibroblasts having been removed from their native in vivo environments and put into the same culture conditions, these two EV proteome profiles remained distinct.

Functional analysis of the TenX proteome identified an enrichment of ECM proteins (collagens), translational proteins (ribosomal proteins) and cytoskeletal proteins (keratins and myosins), which is consistent with the proteome of porcine tendon exosomes [18]. On the other hand FibX distinctly lacked a high abundance of ribosomal proteins, Golgi transport proteins and ATPase ion-transporters but contained significantly higher levels of the collagenase MMP2 than TenX. FibX were also uniquely enriched in the ECM proteins fibulin-1, nidogen-2 and tenascin X, all of which are components of the skeletal muscle ECM [44]; TIMP3, a negative regulator of tumour necrosis factor α autocrine signalling in satellite cells during muscle regeneration [45]; and acetyl-CoA acyl-transferase, a subunit of the mitochondrial trifunctional protein complex and autosomal recessive mutations of this gene causes muscle weakness [46–48]. The presence of these proteins could indicate the mechanisms of interaction between the skeletal muscle fibroblasts and the muscle cell (Fig. 6), an idea that is supported by earlier findings that demonstrated an intimate interplay between different cell types in skeletal muscle through EVs [20]. Further, it has been shown that de-adhesion and adhesion activity is involved in muscle regeneration after heavy mechanical loading [49], suggesting that the intramuscular connective tissue is prepared to coordinate tasks in adaptation and regeneration with the myonuclei of the muscle cell and the satellite cells.

More than half of secreted TGFβ1 are localised to EVs [11] and it is commonly identified in EVs released by cancer associated fibroblasts [8]. Here we report that small EVs released by healthy human tendon fibroblasts also contained a significantly high abundance of TGFβ1 compared to MyoX and FibX. A recent study showed that injection of exosomes isolated from cultures of tendon progenitors into an in vivo collagenase-induced tendinopathy model in rat Achilles increased type I collagen synthesis and improved tissue biomechanics [16]. Another study showed that the ability of tendon exosomes to reprogram mesenchymal stem cells to produce type I collagen could be blocked using a TGFβ inhibitor [19]. Thus, we hypothesise that it is likely that TGFβ1 is also a functional cargo of tendon EVs that may regulate the tendon ECM. Further studies confirming TGFβ localisation in TenX and its specific function are required and these should be performed carefully to exclude activity associated with co-isolated soluble mediators [33]. Together these data suggest that tendon EVs have the potential to induce collagen synthesis, transportation of new proteins for post-translational modifications and ECM assembly in recipient cells (Fig. 6), whereas EVs derived from muscle fibroblasts may maintain the skeletal muscle ECM as well as the low fibrotic potential of healthy muscle fibroblasts [50].

Muscle tissue-derived exosomes are released into the circulation upon exercise and are targeted to the liver [51]. We propose that small EVs produced by tendon cells, however, remain in the tissue under normal conditions and are used to synchronise ECM remodelling via autocrine and paracrine signalling. Rupture of the tissue could lead to the release of tendon EVs into the tissue surroundings, such as the adjacent muscle, potentially being responsible for the initiation of a transient fibrotic response.

Isolation of specific types of EVs is challenging with small samples. Although we were able to identify many exosome-enriched proteins in our samples, the protocol we used, without the inclusion of a density-gradient separation step that produces a higher purity of exosomes at the cost of yield, does not separate out other small vesicles [52, 53]. We observed a large number of ribosomal proteins that are commonly co-isolated with EVs (Supplementary Figure 3d). The role of these ribosomal proteins may include forming protein-RNA complexes inside exosomes and other EVs [54, 55] but it is possible that they are co-isolated as non-EV protein aggregates rather than promiscuous loading [56, 57]. Further investigations of purer exosome isolations in combination with immuno-electron microscopy would establish the location of ribosomal and ECM proteins identified by proteomics.

The proteome profiles revealed the potential functions of fibroblast EVs but it is clear that the EV protein contents capture a snap shot of the cell’s biochemistry at a given time, e.g. the differentiation of myo-progenitors. Therefore EVs could permit long-term monitoring of tissue health in a non-invasive manner in tissues including tendon [58] that cannot be sampled repeatedly. A very recent study demonstrated that exosomes from different sources are characterised by specific combinations of their surface proteins that can be quantified using antibody-based barcoding assay [59]. We identified five membrane proteins that are significantly enriched in tendon EVs (ANTXR2, PMCA4, PTGFRN, syntaxin-4 and syntaxin-7) and one membrane protein that is significantly enriched in muscle fibroblast EVs (CD73) that, hypothetically, could be targeted in combination with other exosome-specific surface proteins in antibody-binding assays to capture themfrom various bodily fluids [29, 30] for analysis. The ability to identify tissue-specific EVs expands the potential of exosomes as biomarker carriers in non-cancerous diseases.

## Conclusions

This study reports, for the first time, comprehensive proteome profiles for small EVs released from healthy human fibroblasts and their potential roles in tissue homeostasis. Our results also demonstrate that with LC-MS-based proteome profiling it was possible to reveal a marked heterogeneity among fibroblast-derived small EVs, indicating shared tasks between EVs in skeletal muscle myoblasts and fibroblast, whereas tendon fibroblast extracellular vesicles demonstrated a potential to be pro-fibrotic in human tendon tissue.

## Supplementary information

**Additional file 1.** Supplementary Data 1. Mass spectrometry data of list of proteins and normalised intensities in all samples.

**Additional file 2.** Supplementary Data 2. Customised R code for iDEP analyses.

**Additional file 3.** Supplementary Data 3. Log transformed intensity list with missing values filled in by imputation.

**Additional file 4.** Supplementary Data 4. Heatmap values for proteins ranked by SD.

**Additional file 5.** Supplementary Data 5. Lists of proteins, fold changes and FDR values from DESeq2 analyses of TenX-MyoX, TenX-FibX and MyoX-FibX comparisons.

**Additional file 6.** Supplementary Data 6. Fold change values of heatmaps from TenX-MyoX, TenX-FibX and MyoX-FibX DESeq2 analyses.

**Additional file 7.** Supplementary Data 7. Functional enrichment analysis results for high abundance proteins in MyoX, TenX and FibX.

**Additional file 8.** Supplementary Data 8. Functional enrichment analysis result for proteins with differential abundance between TenX and FibX.

**Additional file 9.** Supplementary Data 9. Venn diagram outputs.

**Additional file 10 **Supplementary Figure 1. Overview of expansion of muscle tissue and tendon cells for small EV isolation. **a** Illustrative overview of cell isolation and expansion for small EV isolation from differentiating myoblasts, muscle fibroblasts and tendon fibroblasts (*n* = 5 except *n* = 4 for FibX because muscle fibroblasts from prep 5* did not proliferate enough for small EV isolation). **b** Table of information for the five samples used for LC-MS. **c** Transmission electron microscopy images (TEM) of negative-stained EV isolates. Bars, 200 nm. **(D)** Measured diameter of EVs from TEM images. At least 300 measurements for each sample (*n* = 3 biological samples). Supplementary Figure 2. Variances of small EV protein abundance detected by LC/MS. **a** Summary of protein exclusion for statistical analysis by iDEP. **b-c** Distribution of log transformed protein abundances in MyoX, TenX and FibX as detected by LC-MS shown in a box plot (**b**) and a density plot (**c**). Supplementary Figure 3. Characterisation of protein content of isolated EVs. **a-f** Heatmaps showing the levels of protein detected in MyoX, TenX and FibX samples that are proteins enriched in EVs (**a**), cytosolic proteins recovered in EVs via lipid or membrane protein-binding ability (**b**) or promiscuous incorporation (**c**), proteins that have are commonly co-isolated with EVs (**d**), and secreted proteins recovered with EVs (**e**).

## Data Availability

All datasets generated for this study are included in the manuscript/supplementary files including the customised R code for the iDEP workflow. The mass spectrometry data have been deposited to the ProteomeXchange Consortium via the PRIDE partner repository (http://www.ebi.ac.uk/pride/archive/) with the data set identifier PXD018233.

## Abbreviations

ANTXR2: Anthrax toxin receptor 2
APEX1: DNA-(apurinic or apyrimidinic site) lyase
CAFs: Cancer-associated fibroblasts
CHMP: Charged multivesicular body protein
ECM: Extracellular matrix
ESCRT: Endosomal sorting complexes required for transport
EV: Extracellular vesicle
FBLN1: Fibulin-1
FDR: False discovery rate
FibX: Muscle fibroblast-derived extracellular vesicles
GSPT1: G1 to S phase transition 1
HSP: Heatshock protein
iDEP: Integrated web application Differential Expression and Pathway
LC-MS: Liquid chromatography-mass spectrometry
LFQ: Label-free quantitation
MMP2: Matrix metalloproteinase 2
MyoX: Differentiating myoblast-derived extracellular vesicles
PAFAH1B2: Platelet activating factor acetylhydrolase 1b catalytic subunit 2
PCA: Principal component analysis
PMCA4: Plasma membrane Ca^2^^+^−ATPase 4; *ATP2B4*
PTGFRN: Prostaglandin F2 receptor negative regulator
Rab: Ras-associated binding
SD: Standard deviation
TenX: Tendon fibroblast-derived extracellular vesicles
TIMP3: Metalloproteinase inhibitor 3
TGFβ1: Transforming growth factor β1
TNXB: Tenascin-X
