# Supplementary material for: Proteomics identifies differences in fibrotic potential of extracellular vesicles from human tendon and muscle fibroblasts
Source: Cell Commun Signal. 2020 Nov 4;18:177. doi: 10.1186/s12964-020-00669-9 (PMC7641822; doi:10.1186/s12964-020-00669-9)
Supplement: Supplementary file 11 — Additional file 10 Supplementary Figure 1. Overview of expansion of muscle tissue and tendon cells for small EV isolation. a Illustrative overview of cell isolation and expansion for small EV isolation from differentiating myoblasts, muscle fibroblasts and tendon fibroblasts (n = 5 except n = 4 for FibX because muscle fibroblasts from prep 5* did not proliferate enough for small EV isolation). b Table of information for the five samples used for LC-MS. c Transmission electron microscopy images (TEM) of negative-stained EV isolates. Bars, 200 nm. (D) Measured diameter of EVs from TEM images. At least 300 measurements for each sample (n = 3 biological samples). Supplementary Figure 2. Variances of small EV protein abundance detected by LC/MS. a Summary of protein exclusion for statistical analysis by iDEP. b-c Distribution of log transformed protein abundances in MyoX, TenX and FibX as detected by LC-MS shown in a box plot (b) and a density plot (c). Supplementary Figure 3. Characterisation of protein content of isolated EVs. a-f Heatmaps showing the levels of protein detected in MyoX, TenX and FibX samples that are proteins enriched in EVs (a), cytosolic proteins recovered in EVs via lipid or membrane protein-binding ability (b) or promiscuous incorporation (c), proteins that have are commonly co-isolated with EVs (d), and secreted proteins recovered with EVs (e). [file 12964_2020_669_MOESM11_ESM.pdf]

Proteomics Identify Differences in Fibrotic Potential of Fibroblast-Derived Extracellular Vesicles from Tendon and Muscle

Ching-Yan Chloé Yeung[1*], Erwin M. Schoof[2], Michal Tamáš[1], Abigail L. Mackey[1,3], and Michael Kjaer[1].

1. Institute of Sports Medicine Copenhagen, Bispebjerg Hospital, and Center for Healthy Aging, University of Copenhagen, Copenhagen, Denmark.
2. Proteomics Core, Technical University of Denmark, Kongens Lyngby, Denmark.
3. Department of Biomedical Sciences, Faculty of Health and Medical Sciences, University of Copenhagen, Copenhagen, Denmark.

Supplementary Figures

Supplementary Figure 1. Overview of expansion of muscle tissue and tendon cells for small EV isolation.
a Illustrative overview of cell isolation and expansion for small EV isolation from differentiating myoblasts, muscle fibroblasts and tendon fibroblasts for proteomics analysis (n=5 except n=4 for FibX because muscle fibroblasts from prep 5* did not proliferate enough for small EV isolation). **b** Table of information for the five samples used for LC-MS. **c** Transmission electron microscopy images (TEM) of negative-stained EV isolates. Bars, 200 nm. **d** Measured diameter of EVs from TEM images. At least 300 measurements for each sample (n=3 biological (patient) samples).

Supplementary Figure 2. Variances of small EV protein abundance detected by LC/MS.
a Summary of protein exclusion for statistical analysis by iDEP. **b-c** Distribution of log transformed protein abundances in MyoX, TenX and FibX as detected by LC-MS shown in a box plot (**b**) and a density plot (**c**).

Supplementary Figure 3. Characterisation of protein content of isolated EVs.
a-e Heatmaps showing the levels of protein detected in MyoX, TenX and FibX samples that are proteins enriched in EVs (**a**), cytosolic proteins recovered in EVs via lipid or membrane protein-binding ability (**b**) or promiscuous incorporation (**c**), proteins that have are commonly co-isolated with EVs (**d**), and secreted proteins recovered with EVs (**e**).

Supplementary Data Files

Supplementary Data 1. Proteomics read out.

Supplementary Data 2. Customised R code for iDEP analyses.

Supplementary Data 3. Log transformed intensity list with missing values filled in by imputation.

Supplementary Data 4. Heatmap values for proteins ranked by SD.

Supplementary Data 5. Lists of proteins, fold changes and FDR values from DESeq2 analyses of TenX-MyoX, TenX-FibX and MyoX-FibX comparisons.

Supplementary Data 6. Fold change values of heatmaps from TenX-MyoX, TenX-FibX and MyoX-FibX DESeq2 analyses.

Supplementary Data 7. Enrichment analysis results for high abundance proteins in MyoX, TenX and FibX.

Supplementary Data 8. Enrichment analysis result for proteins with differentially abundance between TenX and FibX.

Supplementary Data 9. Venn diagram outputs.

Yeung et al. – Supplementary Figure 1

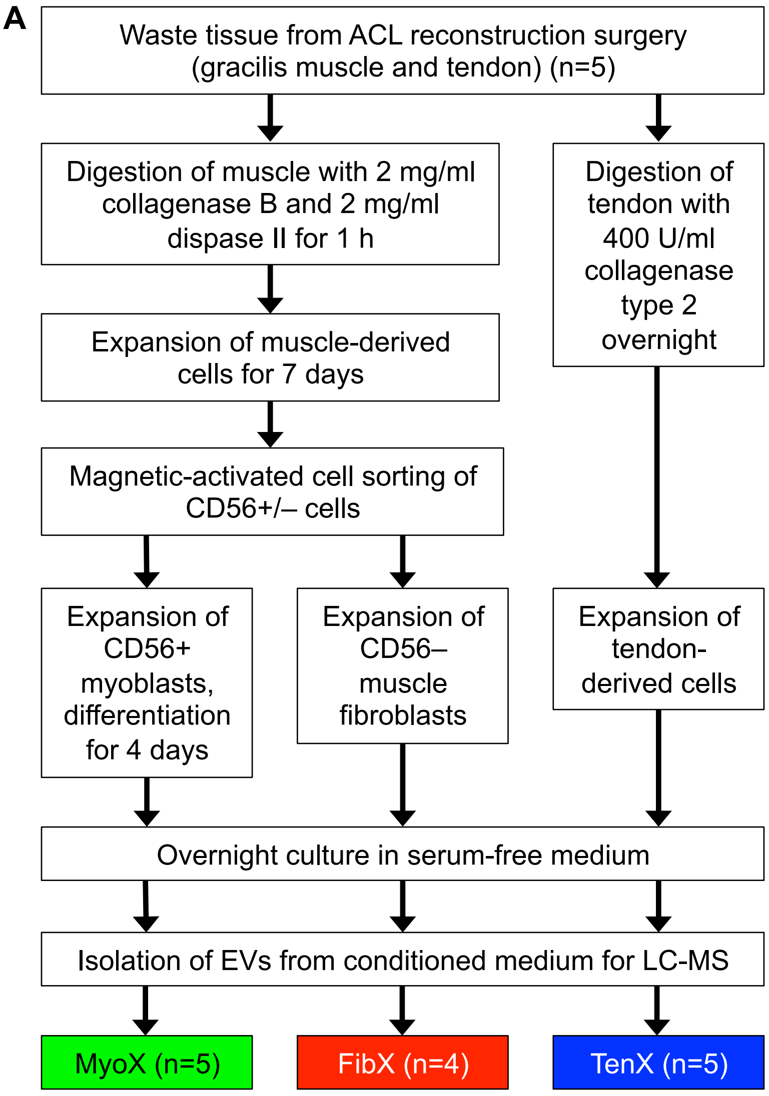

B

LC-MS Sample	Sex	Age
1	F	29
2	M	27
3	F	26
4	M	24
5*	M	42

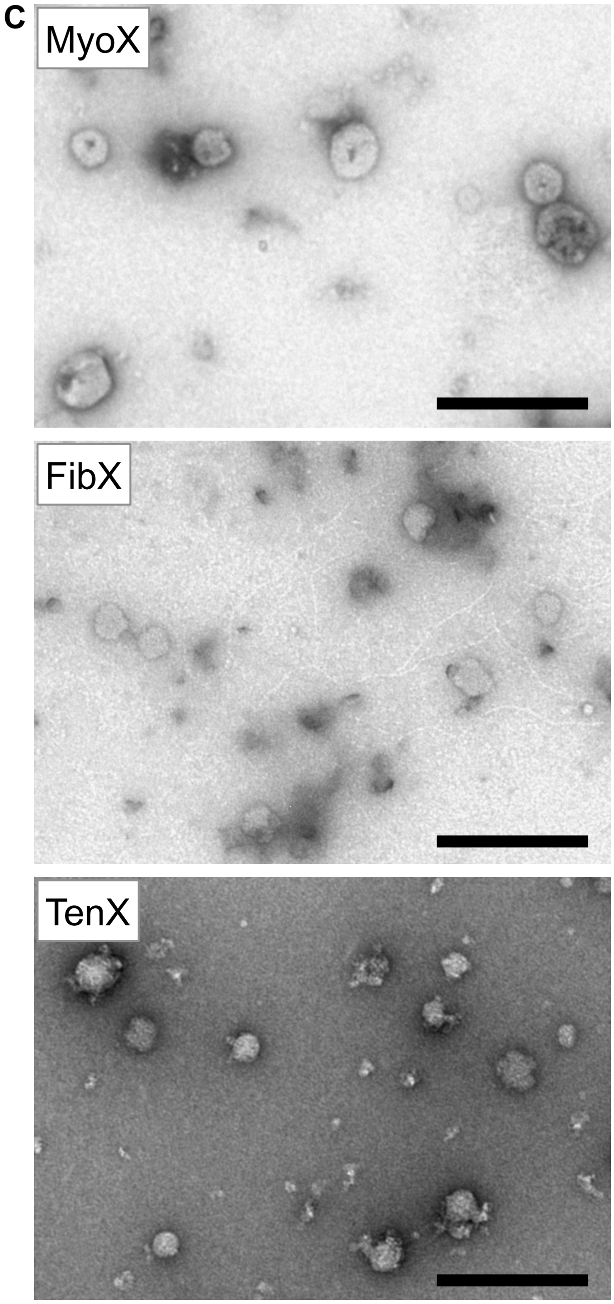

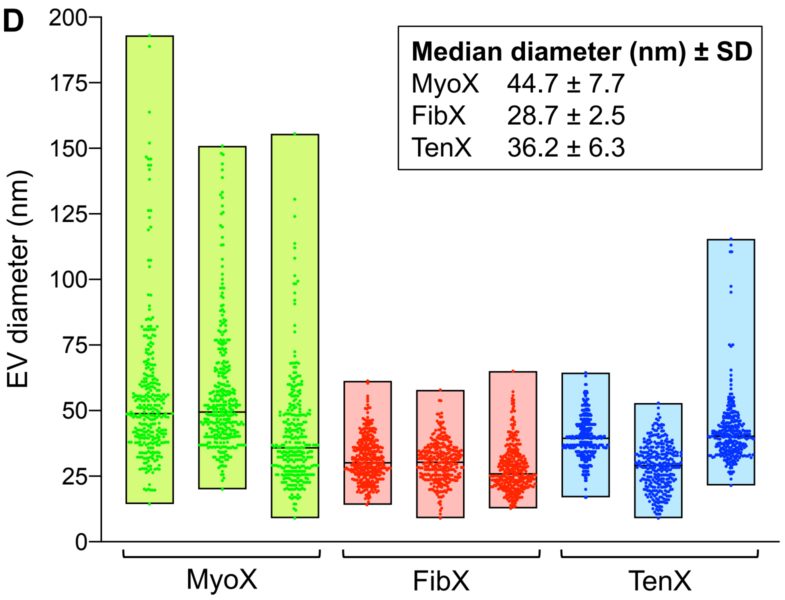

Supplementary Figure 1. Overview of expansion of muscle tissue and tendon cells for small EV isolation. **a** Illustrative overview of cell isolation and expansion for small EV isolation from differentiating myoblasts, muscle fibroblasts and tendon fibroblasts for proteomics analysis (n=5 except n=4 for FibX because muscle fibroblasts from prep 5* did not proliferate enough for small EV isolation). **b** Table of information for the five samples used for LC-MS. **c** Transmission electron microscopy images (TEM) of negative-stained EV isolates. Bars, 200 nm. **d** Measured diameter of EVs from TEM images. At least 300 measurements for each sample (n=3 biological samples).

Yeung et al. – Supplementary Figure 2

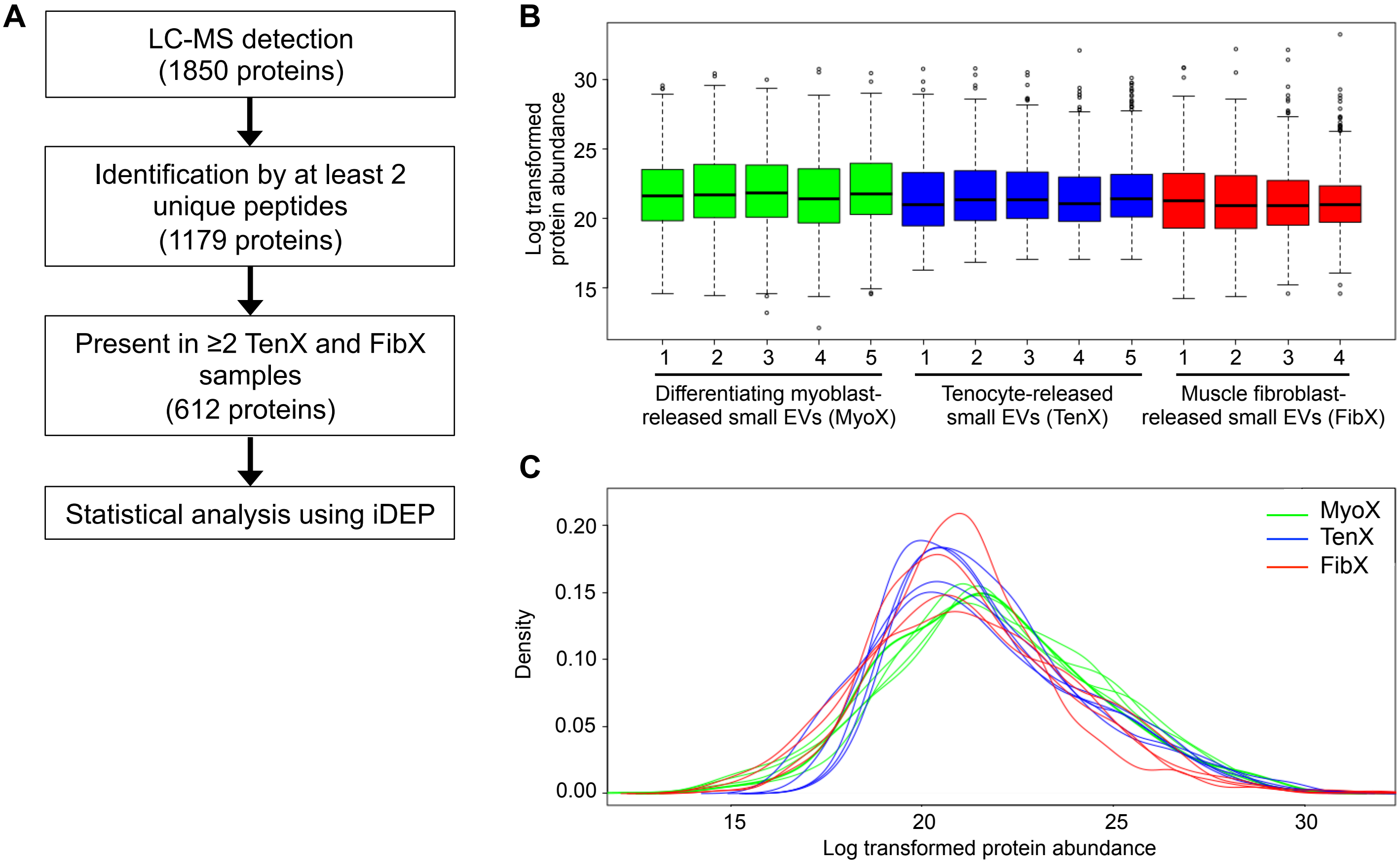

Supplementary Figure 2. Variances of EV protein abundance detected by LC/MS.
a Summary of protein exclusion for statistical analysis by iDEP. **b-c** Distribution of log transformed protein abundances in MyoX, TenX and FibX as detected by LC-MS shown in a box plot (**b**) and a density plot (**c**).

Yeung et al. – Supplementary Figure 3

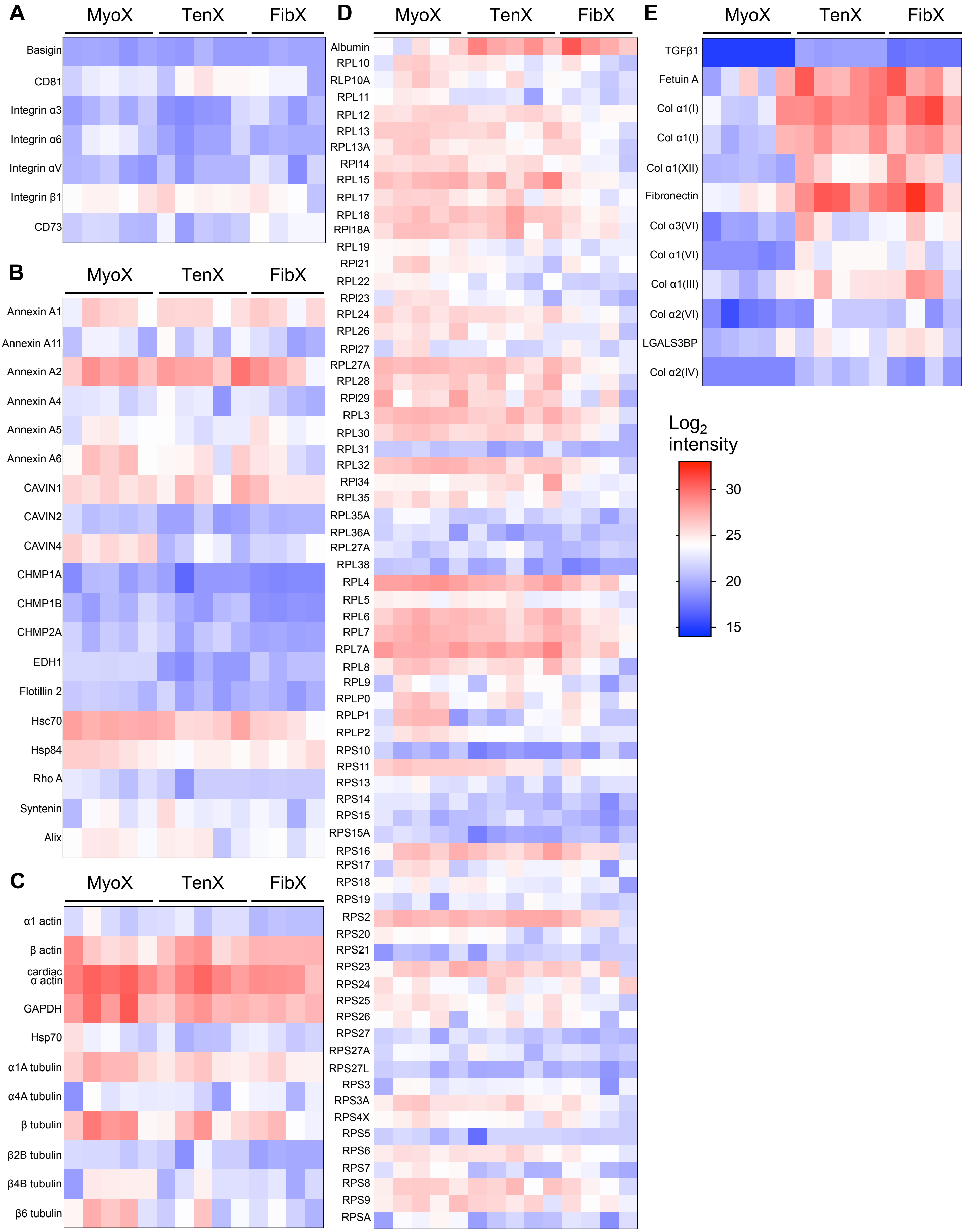

Supplementary Figure 3. Characterisation of protein content of isolated EVs.
a-e Heatmaps showing the levels of protein detected in MyoX, TenX and FibX samples that are proteins enriched in EVs (**a**), cytosolic proteins recovered in EVs via lipid- or membrane protein-binding ability (**b**) or promiscuous incorporation (**c**), proteins that have are commonly co-isolated with EVs (**d**), and and secreted proteins recovered with EVs (**e**).
